# Supplementary material for: Left Atrial Thrombus in Atrial Fibrillation/Flutter Patients in Relation to Anticoagulation Strategy: LATTEE Registry
Source: J Clin Med. 2022 May 11;11(10):2705. doi: 10.3390/jcm11102705 (PMC9143266; doi:10.3390/jcm11102705)
Supplement: Supplementary file 1 [file jcm-11-02705-s001.zip › jcm-1668961-supplementary.pdf]

Supplemental material

Left atrial thrombus in atrial fibrillation/flutter patients in relation to anticoagulation strategy: results from the LATTEE registry

Running title: Left atrial thrombus in atrial fibrillation/flutter

Agnieszka Kapłon-Cieślicka[a,b], Monika Gawałko[b-d], Monika Budnik[a,b], Beata Uziębło-Życzkowska[e], Paweł Krzesiński[a,e], Katarzyna Starzyk[f], Iwona Gorczyca[f], Ludmiła Daniłowicz-Szymanowicz[g], Damian Kaufmann[g], Maciej Wójcik[a,h], Robert Błaszczyk[h], Jarosław Hiczkiewicz[i,j], Katarzyna Łojewska[j], Katarzyna Mizia-Stec[a,k], Maciej T. Wybraniec[1,k], Katarzyna Kosmalska[l], Marcin Fijałkowski[1,m], Anna Szymańska[n], Mirosław Dłużniewski[n], Maciej Haberka[a,o], Michał Kucio[o], Błażej Michalski[a,p], Karolina Kupczyńska[a,p], Anna Tomaszuk-Kazberuk[a,r], Katarzyna Wilk-Śledziewska[r], Renata Wachnicka-Truty[s], Marek Koziński[a,s], Paweł Burchardt[1,t], Piotr Scisło[b], Radosław Piątkowski[b], Janusz Kochanowski[b], Grzegorz Opolski[b], Marcin Grabowski[b]

[a] "Club 30", Polish Cardiac Society, Poland
[b] 1st Department of Cardiology, Medical University of Warsaw, Warsaw, Poland
[c] Institute of Pharmacology, West German Heart and Vascular Centre, University Duisburg-Essen, Germany
[d] Department of Cardiology, Maastricht University Medical Centre and Cardiovascular Research Institute Maastricht, Maastricht, The Netherlands
[e] Department of Cardiology and Internal Diseases, Military Institute of Medicine, Warsaw, Poland
[f] 1st Clinic of Cardiology and Electrotherapy, Świętokrzyskie Cardiology Centre, Kielce, Poland
[g] Department of Cardiology and Electrotherapy, Medical University of Gdańsk, Gdańsk, Poland
[h] Department of Cardiology, Medical University of Lublin, Lublin, Poland
[i] Collegium Medicum, University of Zielona Góra, Poland
[j] Clinical Department of Cardiology, Nowa Sól Multidisciplinary Hospital, Nowa Sól, Poland
[k] 1st Department of Cardiology, School of Medicine in Katowice, Medical University of Silesia, Katowice, Poland
[l] Department of Cardiology, St Vincent Hospital, Gdynia, Poland
[m] 1st Department of Cardiology, Medical University of Gdansk, Gdańsk, Poland
[n] Department of Heart Diseases, Postgraduate Medical School, Warsaw, Poland
[o] Department of Cardiology, School of Health Sciences, Medical University of Silesia, Katowice, Poland
[p] Department of Cardiology, Medical University of Lodz, Łodz, Poland
[r] Department of Cardiology, Medical University of Bialystok, Białystok, Poland
[s] Department of Cardiology and Internal Medicine, Medical University of Gdańsk, Gdynia, Poland
[t] Department of Hypertension, Angiology, and Internal Medicine, Poznan University of Medical Sciences, Poznan, Poland

***Corresponding author:**
Monika Gawałko, MD, PhD
1st Department of Cardiology, Medical University of Warsaw
1a Banacha St., Warsaw 02-097, Poland
T: +48 22 599 29 58 | F: +48 22 599 19 57 | Mail: mgawalko@wum.edu.pl

Table S1. Baseline characteristics of patients with atrial fibrillation/atrial flutter undergoing transesophageal echocardiography before cardioversion or ablation.

Variable	Overall (n=3109)	Chronic OAC (n=2753)	No OAC (n=307)	p[1]
**Demographics**				
Age(years)	67 [59-73] n=3104	67 [60-73] n=2749	66 [57-74] n=306	0.44
Age≥75 years	668/3105(22%)	579/2749(21%)	73/306 (24%)	0.27
Female sex	1145/3108 (37%)	1022/2753 (37%)	105/306 (34%)	0.35
BMI(kg/m^2)	29 [26-33] n=2857	29 [26-33] n=2570	29 [25-32] n=287	**<0.01**
**Indications for TEE**				
Direct current cardioversion for AF/AFl	1595/3063 (52%)	1337/2711 (49%)	218/304 (72%)	**<0.01**
AF/AFl ablation	1468/3063 (48%)	1374/2711 (51%)	86/304 (28%)	**<0.01**
**AF/AFl type**				
AF	2733/3109 (88%)	2453/2751 (89%)	240/307 (78%)	**<0.01**
AFl	472/3109 (15%)	389/2751 (14%)	73/307 (24%)	**<0.01**
AF/AFl paroxysmal	1280/3099 (41%)	1127/2746 (41%)	133/304 (44%)	0.39
AF/AFl persistent	1548/3099 (50%)	1369/2746 (50%)	151/304 (50%)	0.95
AF/AFl long-standing persistent	271/3099 (8.7%)	250/2746 (9.1%)	20/304 (6.6%)	0.17
**Comorbidities**				
Hypertension	2366/3107 (76%)	2121/2751 (77%)	205/307 (68%)	**<0.01**
Heart failure	1336/3100 (43%)	1156/2744 (42%)	157/307 (51%)	**<0.01**
Mechanical valve prosthesis	73/3105 (2.4%)	71/2749 (2.6%)	1/307 (0.3%)	**<0.01**
Biological valve prosthesis (including TAVI)	54/3105 (1.7%)	48/2749 (1.8%)	6/307 (2.0%)	0.82
Vascular disease	1066/3107 (34%)	948/2751 (34%)	97/307 (32%)	0.34
Previous stroke	235/3107 (7.6%)	211/2751 (7.7%)	21/307 (6.8%)	0.73
Previous ischemic stroke/ TIA/systemic embolism	313/3102 (10%)	285/2746 (10%)	25/307 (8.1%)	0.23
Previous bleeding	131/3107 (4.2%)	99/2751 (3.6%)	28/307 (9.1%)	**<0.01**
Diabetes mellitus	774/3107 (25%)	679/2751 (25%)	70/307 (23%)	0.49
GFR(mL/min)	81 [63-102] n=2790	82 [63-102] n=2470	78 [60-100] n=278	**0.046**
GFR<50 mL/min	310/2790 (11%)	265/2470 (11%)	40/278 (14%)	0.07
COPD	161/3107 (5.2%)	136/2751 (4.9%)	23/307 (7.5%)	0.08
Anemia[2]	484/3002 (16%)	414/2661 (16%)	58/294 (20%)	0.07
**Thromboembolic risk and indications to chronic OAC**				
CHA_2DS_2-VASc score	3 [2-4] n=3088	3 [2-4] n=2733	3 [1-4] n=306	0.17
Class I indications to OAC[3]	2295/3089 (74%)	2046/2734 (75%)	210/306 (69%)	**0.03**
-moderate-severe MS or mechanical valve prosthesis	89/3089 (2.9%)	87/2734 (3.2%)	1/306 (0.3%)	**<0.01**
Class IIa indications to OAC[4]	526/3089 (17%)	461/2734 (17%)	57/306 (19%)	0.47

No indications to chronic OAC[5]	267/3089 (8.6%)	227/2734 (8.3%)	39/306 (13%)	**0.02**
**Antithrombotic treatment other than OAC**				
Heparin(periprocedural):	215/3074 (7.0%)	109/2744 (4.0%)	100/283 (35%)	**<0.01**
-heparin≥2 days	66/3059 (2.2%)	23/2742 (8.4%)	42/272 (15%)	**<0.01**
Antiplatelets	323/3107 (10%)	238/2753 (8.7%)	79/307 (26%)	**<0.01**
**Transesophageal echocardiography**				
Left atrial appendage emptying velocity(cm/s)	38 [26-53] n=2644	39 [26-53] n=2336	36 [25-53] n=271	0.56
SEC	808/3074 (26%)	688/2721 (25%)	98/305 (32%)	**0.01**
Left atrial thrombus	250/3109 (8.0%)	200/2753 (7.3%)	45/307 (15%)	**<0.01**

Legend:

[1] p value for difference between patients on chronic OAC vs no OAC

[2] hemoglobin <12g/dl for female <13g/dl for male

[3] CHA2DS2-VASc score >=2 for men and >=3 for women or moderate-severe MS or mechanical valve prosthesis

[4] CHA2DS2-VASc score 1 for men and 2 for women

[5] CHA2DS2-VASc score 0 for men and 1 for women

Abbreviations: AF, atrial fibrillation; BMI, body mass index; COPD, chronic obstructive pulmonary disease; CRT, cardiac resynchronization therapy; EF, ejection fraction; GFR, glomerular ejection fraction; ICD, implantable cardioverter defibrillator; OAC, oral anticoagulant; MS, mitral stenosis; NYHA, New York Heart Association; PM, pacemaker; SEC, spontaneous echocardiographic contrast; TAVI, transcatheter aortic valve implantation; TIA, transient ischemic attack; VTE, venous thromboembolism

Table S2. Baseline characteristics of all hospitalized atrial fibrillation patients depending on presence of left atrial thrombus (n=3109).

Variable	Without LA thrombus (n=2859)	With LA thrombus (n=250)	p[1]
**Demographics**			
Age (years)	67 [59-73] *n=2854*	72 [64-78]	**<0.01**
Age ≥75 years	580/2854 (20%)	88/250 (35%)	**<0.01**
Female sex	1056/2854 (37%)	89/250 (36%)	0.73
BMI (kg/m^2)	29 [26-33] *n=2275*	30 [26-32] *n=179*	0.09
**Indications for TEE**			
Direct current cardioversion for AF/AFl	1398/2815 (50%)	197/248 (79%)	**<0.01**
AF/AFl ablation	1417/2815 (50%)	51/248 (21%)	**<0.01**
**AF/AFl type**			
AF	2513//2859 (88%)	219/250 (88%)	0.92
AFl	437/2859 (15%)	35/250 (14%)	0.65
AF/AFl paroxysmal	1247/2849 (44%)	33/250 (13%)	**<0.01**
AF/AFl persistent	1365/2849 (48%)	183/250 (73%)	**<0.01**
AF/AFl long-standing persistent	237/2849 (8.3%)	34/250 (14%)	**<0.01**
**Comorbidities**			
Hypertension	2171/2859 (76%)	195/248 (79%)	0.34
Heart failure	1165/2852 (41%)	17/2481 (69%)	<**0.01**
Mechanical valve prosthesis	64/2856 (2.2%)	9/249 (3.6%)	0.19
Biological valve prosthesis (including TAVI)	47/2856 (1.7%)	7/249 (2.8%)	0.20
Vascular disease	949/2859 (33%)	118/249 (47%)	**<0.01**
Coronary artery disease	811/2859 (29%)	94/248 (38%)	**<0.01**
Peripheral artery disease	149/2859 (5.2%)	26/248 (10%)	**<0.01**
Previous stroke	206/2859 (7.2%)	29/248 (12%)	**0.02**
Previous ischemic stroke/TIA/systemic embolism	278/2855 (9.7%)	35/247 (14%)	**0.04**
Previous bleeding	114/2859 (4.0%)	17/248 (6.9%)	**0.046**
Previous hemorrhagic stroke	14/2855 (0.5%)	3/247 (1.2%)	0.15
Diabetes mellitus	683/2859 (24%)	91/248 (37%)	**<0.01**
GFR (mL/min)	82 [64-103]<br>n=2571	74 [51-93]<br>n=219	**<0.01**
GFR <50 mL/min	258 (10%)<br>n=2571	52 (24%)<br>n=219	**<0.01**
COPD	145/2859 (5.1%)	16/248 (6.5%)	0.37
Anemia[3]	431/2773 (16%)	53/229 (23%)	**<0.01**
**Thromboembolic risk and indications to chronic OAC**			
CHA_2DS_2-VASc score	3 [2-4] *n=2843*	4 [3-5] *n=245*	**<0.01**
Class I indications to OAC[4]	2070/2844 (73%)	226/245 (92%)	**<0.01**

- moderate/ severe MS or mechanical valve prosthesis	75/2844 (2.6%)	14/245 (5.7%)	**0.01**
Class IIa indications[5]	510/2844 (18%)	16/245 (6.5%)	**<0.01**
No indications to chronic OAC[6]	264/2844 (9.3%)	3/245 (1.2%)	**<0.01**
**Antithrombotic therapy**			
Chronic OAC	2553/2857 (89%)	200/250 (80%)	**<0.01**
No OAC	262/2857 (9.2%)	45/250 (18%)	**<0.01**
Heparin (periprocedural):	186/2835 (6.6%)	29/239 (12%)	**<0.01**
- heparin ≥2 days	50/172 (29%)	16/28 (57%)	**<0.01**
Antiplatelets	282/2857 (9.9%)	41/240 (17%)	**<0.01**
**Transesophageal echocardiography**			
Left atrial appendage emptying velocity (cm/s)	40 [28-55] *n=2437*	20 [16-27] *n=207*	**<0.01**
SEC	616/2828 (22%)	192/246 (78%)	**<0.01**

Legend:

[1] p value for difference between patients on chronic OAC vs no OAC

[2] p value for difference between rivaroxaban, dabigatran and apixaban

[3] hemoglobin <12g/dl for female <13g/dl for male

[4] CHA2DS2-VASc score >=2 for men and >=3 for women or moderate-severe MS or mechanical valve prosthesis

[5] CHA2DS2-VASc score 1 for men and 2 for women

[6] CHA2DS2-VASc score 0 for men and 1 for women

Abbreviations: See **Table 1;** LA, left atrial

Table S3. Comparison of chronic OAC groups after propensity score weighting.

**A.** VKA vs. NOAC			
**Variable**	**VKA (n=491)**	**NOAC (n=491)**	**p value**
**Variables used for propensity score weighting**			
Age ≥65 years	318 (65%)	325 (66%)	0.69
Females	196 (40%)	195 (40%)	1.00
Heart failure	254 (52%)	255 (52%)	1.00
Hypertension	385 (78%)	396 (81%)	0.43
Vascular disease	193 (39%)	176 (36%)	0.29
Diabetes	141 (29%)	136 (28%)	0.78
Ischemic stroke/TIA/systemic embolism	56 (11%)	45 (9.2%)	0.29
GFR <50 mL/min	117 (24%)	105 (21%)	0.40
Left atrial appendage emptying velocity (cm/s)	27 [17-41]	28 [18-41]	0.77
**Results**			
SEC	165 (34%)	133 (27%)	**0.03**
Dense SEC	82 (17%)	32 (6.5%)	**<0.01**
Left atrial thrombus	64 (13%)	38 (7.5%)	**<0.01**
**B.** Rivaroxaban vs. dabigatran (standard doses)			
**Variable**	**Rivaroxaban standard dose (n=677)**	**Dabigatran standard dose (n=677)**	**p value**
**Variables used for propensity score weighting**			
Age ≥65 years	299 (44%)	314 (46%)	0.44
Females	217 (32%)	220 (33%)	0.91
Heart failure	246 (36%)	224 (33%)	0.23
Hypertension	518 (77%)	502 (74%)	0.34
Vascular disease	205 (30%)	187 (28%)	0.31
Diabetes	162 (24%)	153 (23%)	0.61
Ischemic stroke/TIA/systemic embolism	58 (8.6%)	59 (8.7%)	1.00
GFR <50 mL/min	79 (12%)	97 (14%)	0.17
Left atrial appendage emptying velocity (cm/s)	40 [23-58]	40 [26-57]	0.35
**Results**			
SEC	134 (20%)	124 (18%)	0.53
Dense SEC	36 (5.3%)	39 (5.8%)	0.81
Left atrial thrombus	25 (3.7%)	27 (4.0%)	0.89
**C.** Rivaroxaban vs. apixaban (standard doses)			
**Variable**	**Rivaroxaban standard dose (n=275)**	**Apixaban standard dose (n=275)**	**p value**
**Variables used for propensity score weighting**			
Age ≥65 years	185 (67%)	184 (67%)	1.00
Females	123 (45%)	121 (44%)	0.93
Heart failure	125 (45%)	122 (44%)	0.86
Hypertension	219 (80%)	212 (77%)	0.53
Vascular disease	103 (38%)	103 (38%)	1.00
Diabetes	62 (23%)	71 (26%)	0.43

Ischemic stroke/TIA/systemic embolism	27 (9.8%)	29 (11%)	0.89
GFR <50 mL/min	46 (17%)	45 (16%)	1.00
Left atrial appendage emptying velocity (cm/s)	34 [21-48]	35 [21-50]	0.50
**Results**			
SEC	77 (28%)	69 (25%)	0.50
Dense SEC	18 (6.6%)	19 (6.9%)	1.00
Left atrial thrombus	13 (4.7%)	23 (8.4%)	0.12

D. Dabigatran vs. apixaban (standard doses)

Variable	Dabigatran standard dose (n=275)	Apixaban standard dose (n=275)	p value
**Variables used for propensity score weighting**			
Age ≥65 years	191 (69%)	184 (70%)	0.58
Females	112 (41%)	121 (44%)	0.49
Heart failure	119 (43%)	122 (44%)	0.86
Hypertension	195 (71%)	212 (77%)	0.12
Vascular disease	101 (37%)	103 (37%)	0.93
Diabetes	76 (28%)	71 (26%)	0.70
Ischemic stroke/TIA/systemic embolism	18 (6.5%)	29 (11%)	0.13
GFR <50 mL/min	35 (13%)	45 (16%)	0.28
Left atrial appendage emptying velocity (cm/s)	37 [23-50]	35 [21-50]	0.15
**Results**			
SEC	60 (22%)	69 (25%)	0.42
Dense SEC	29 (11%)	19 (6.9%)	0.17
Left atrial thrombus	19 (6.9%)	23 (8.4%)	0.63

Abbreviations: See **Table 1**

Figure S1. Atrial fibrillation/flutter patients with left atrial thrombus on transesophageal echocardiography:

A. Overall, in relation to presence of oral anticoagulation (n=250)

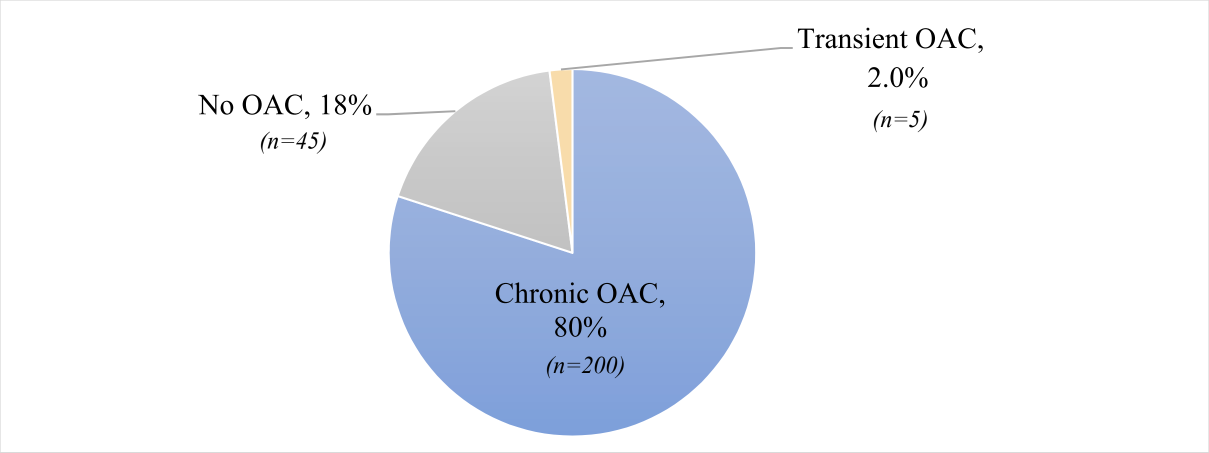

B. Patients on chronic oral anticoagulation (n=200)

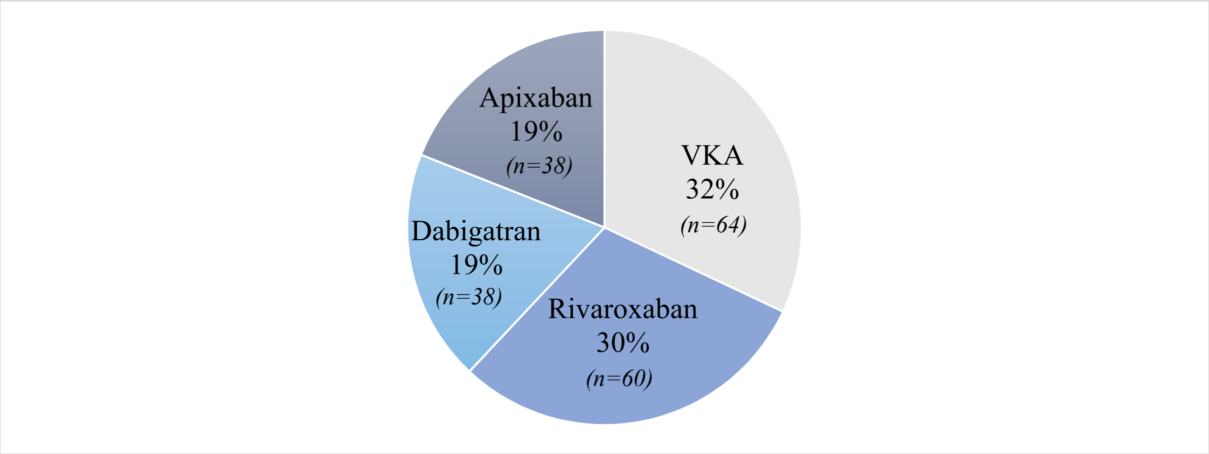

C. Overall, in relation to indications to chronic anticoagulation (n=245)

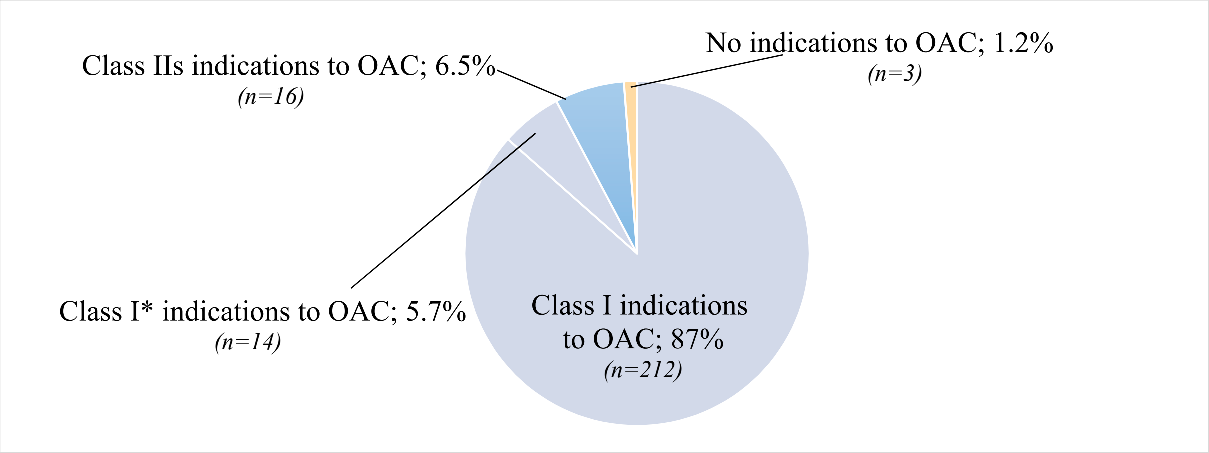

* patients with moderate/severe mitral stenosis or mechanical valve prosthesis

Legend:

No indications to chronic anticoagulation: CHA_2DS_2-VASc 0 (if male) and 1 (if female).

Class IIa indications to chronic anticoagulation: CHA_2DS_2-VASc 1 (if male) and 2 (if female).

Class I indications to chronic anticoagulation: CHA_2DS_2-VASc >=2 (if male) and >=3 (if female) or moderate-severe mitral stenosis or mechanical valve prosthesis

Figure S2. Prevalence of left atrial thrombus in patients on chronic oral anticoagulation (OAC) in relation to indications to OAC.

A. Overall (n=2646)

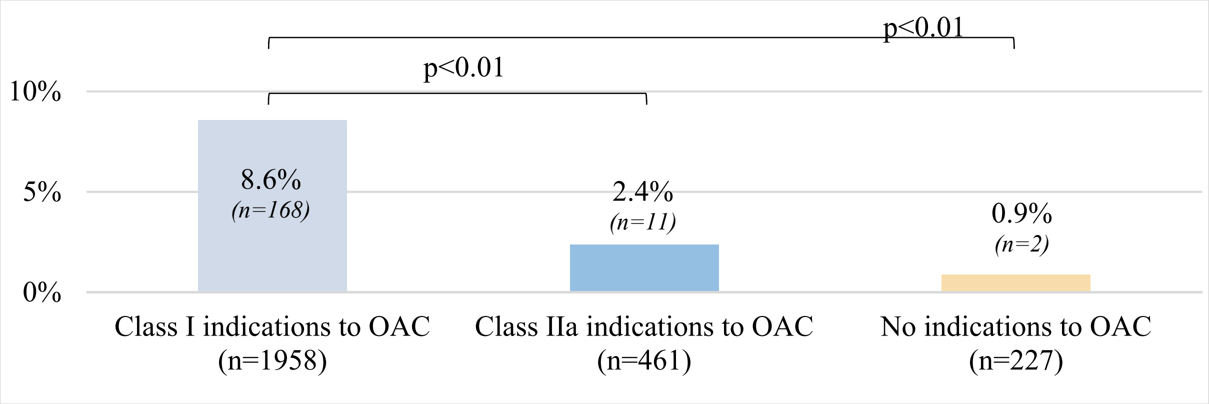

Only p-values of 0.05 or lower were shown for group comparisons

B. In patients with class I indications to chronic anticoagulation (n=1958)

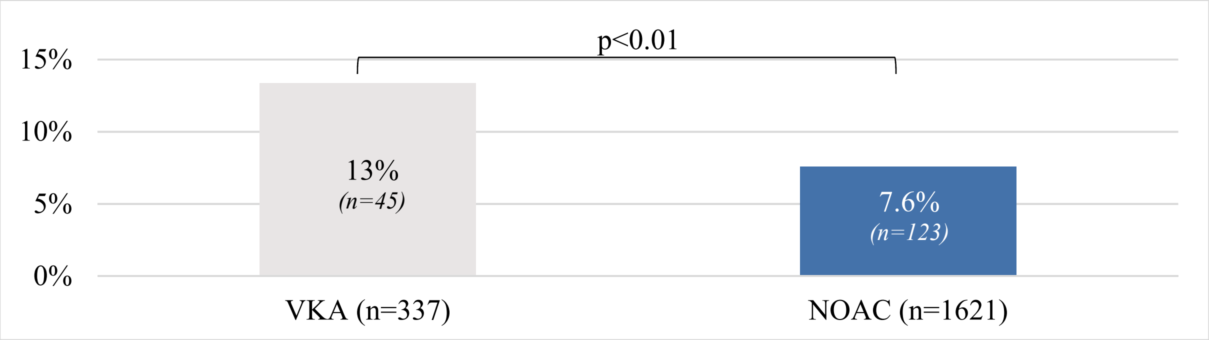

C. In patients with class IIa indications do chronic anticoagulation (n=461)

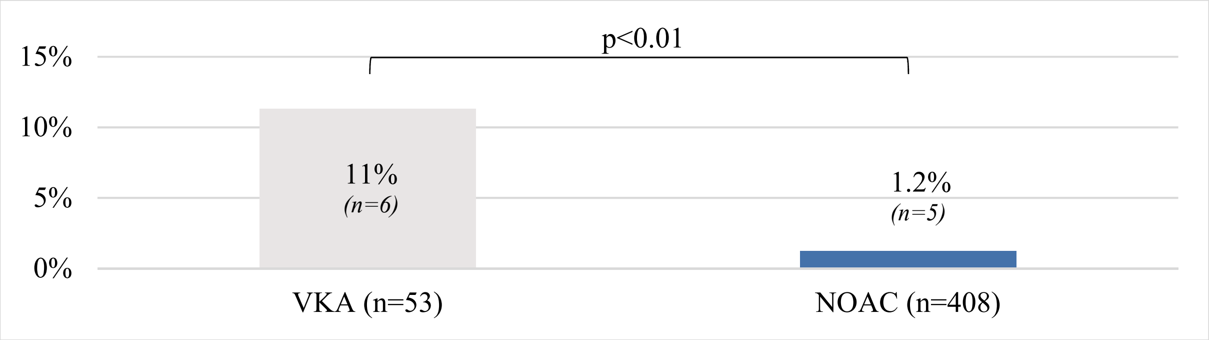

D. In patients with no indications to chronic anticoagulation (n=227)

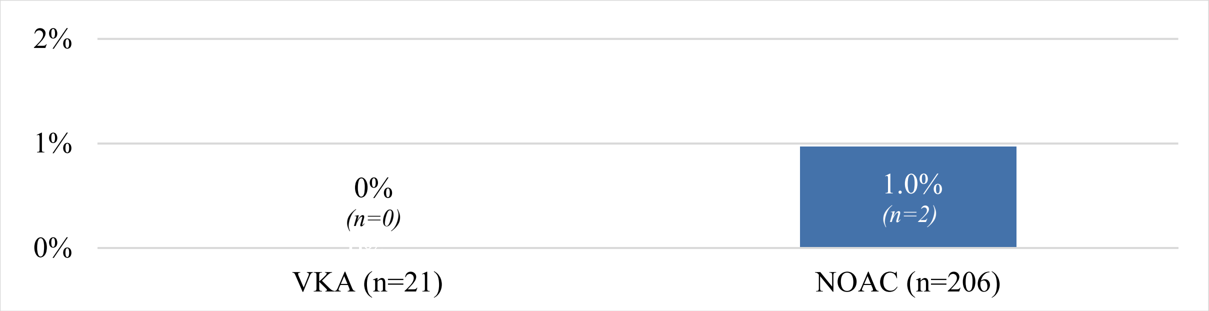

Only p-values of 0.05 or lower were shown for group comparisons

Legend:

No indications to chronic anticoagulation: CHA_2DS_2-VASc 0 (if male) and 1 (if female).

Class IIa indications to chronic anticoagulation: CHA_2DS_2-VASc 1 (if male) and 2 (if female).

Class I indications to chronic anticoagulation: CHA_2DS_2-VASc >=2 (if male) and >=3 (if female)

Patients with moderate-severe mitral stenosis or mechanical valve prosthesis were excluded

Abbreviations: See **Table 1; Figure2**
